# CpG Island Methylator Phenotype and Prognosis of Colorectal Cancer in Northeast China

**DOI:** 10.1155/2014/236361

**Published:** 2014-08-28

**Authors:** Xia Li, Fulan Hu, Yibaina Wang, Xiaoping Yao, Zuoming Zhang, Fan Wang, Guizhi Sun, Bin-Bin Cui, Xinshu Dong, Yashuang Zhao

**Affiliations:** ^1^Department of Epidemiology, Public Health College, Harbin Medical University, Harbin, Heilongjiang 150081, China; ^2^Department of Surgery, Cancer Hospital of Harbin Medical University, Harbin, Heilongjiang 150081, China

## Abstract

*Purpose*. To investigate the association between CpG island methylator phenotype (CIMP) and the overall survival of sporadic colorectal cancer (CRC) in Northeast China. *Methods*. 282 sporadic CRC patients were recruited in this study. We selected *MLH1*, *MGMT*, *p16*, *APC*, *MINT1*, *MINT31*, and *RUNX3* as the CIMP panel markers. The promoter methylation was assessed by methylation sensitive high resolution melting (MS-HRM). Proportional hazards-regression models were fitted with computing hazard ratios (HR) and the corresponding 95% confidence intervals (95% CI). *Results*. 12.77% (36/282) of patients were CIMP-0, 74.1% (209/282) of patients were CIMP-L, and 13.12% (37/282) of patients were CIMP-H. The five-year survival of the 282 CRC patients was 58%. There was significant association between *APC* gene promoter methylation and CRC overall survival (HR = 1.61; 95% CI: 1.05–2.46; *P* = 0.03). CIMP-H was significantly associated with worse prognosis compared to CIMP-0 (HR = 3.06; 95% CI: 1.19–7.89; *P* = 0.02) and CIMP-L (HR = 1.97; 95% CI: 1.11–3.48; *P* = 0.02), respectively. While comparing with the combine of CIMP-L and CIMP-0 (CIMP-L/0), CIMP-H also presented a worse prognosis (HR = 2.31; 95% CI: 1.02–5.24; *P* = 0.04). *Conclusion*. CIMP-H may be a predictor of a poor prognosis of CRC in Northeast China patients.

## 1. Introduction 

Colorectal cancer (CRC) is one of the most common malignancies, representing the third most common cancer in men and the second in women worldwide [1]. In 2012, about 694,000 deaths from CRC were estimated in the world, which account for 8.5% of all cancer deaths, making it the fourth most common cause of death from cancer [2]. Although the relative 5-year survival of CRC increased in Europe during 1995–2007 [3], it was only about 30%–65% worldwide [4]. Anatomic and pathological staging are still the most accurate predictors of CRC prognosis until now. Therefore, novel molecular prognostic markers for colorectal cancer are needed for the accurate prediction of prognosis.

DNA methylation of tumor suppressor genes leading to transcriptional inactivation has been identified as an important mechanism in human carcinogenesis [5, 6]. CpG island methylation phenotype (CIMP), characterized by the extensive hypermethylation of multiple CpG islands, is currently recognized as one of the major mechanisms in the colorectal carcinogenesis [7, 8]. Compared to CIMP-low/negative CRCs, CIMP-high/positive CRCs have distinct clinicopathological and molecular profiles such as older age, female gender, proximal tumor location, poorly differentiated or mucinous histology, and high rates of MSI and BRAF mutation [9, 10]. Most of these clinicopathological and molecular features of CIMP-high tumors overlap with sporadic MSI cancers; patients with MSI CRCs have a better prognosis [11]; therefore, CIMP statuses are supposed to influence the prognosis of CRC.

Until now, more than 24 papers published focused on the CIMP status and CRC prognosis. Although CIMP-H patients have been reported to be related to poor prognosis of CRC in at least eight studies [5, 6, 12–17], 15 studies reported a null association between CIMP-H and CRC prognosis or even noted a better prognosis [18–32]. One study reported a significant association between CIMP-H and colon cancer specific mortality [33]. Because of the unclear biology cause of CIMP, discrepancy in the methylation markers, and the criteria for CIMP, it still cannot be concluded whether significant association between CIMP-H and CRC prognosis existed. Moreover, no study in China has been published in this aspect. Therefore, we conducted the study to evaluate the association between CIMP status and the prognosis of CRC in Northeast China.

## 2. Methods

### 2.1. Study Participants

After obtaining informed written consent from study subjects and approval from Institutional Research Board of Harbin Medical University, we identified CRC patients who underwent surgery at the Cancer Hospital of Harbin Medical University, without preselection and based on pathologic diagnosis alone. Tumor staging was based on the TNM staging system of the American Joint Committee on Cancer [34]. The histological subtypes were classified using the World Health Organization (WHO) criteria [35]. Patients with neuroendocrine carcinoma, malignant melanoma, non-Hodgkin's lymphoma, gastrointestinal stromal tumors, and metastatic colorectal carcinoma were excluded. Patients who had no family history of CRC regardless of the onset age were categorized as sporadic CRC. From June 1, 2004, to January 1, 2008, 453 primary sporadic CRC patients were recruited, and tumor tissue samples from 282 patients were available for the current study.

We followed patients until March 2012 or death. After surgery, the clinical data of patients were collected based on the medical records for analyses, which included age at diagnosis, tumor location, and pathological diagnosis. During follow-up, chemotherapy and radiotherapy protocols were obtained. Meanwhile, we obtained information about disease progression, recurrence, and the date and cause of death (if deceased).

### 2.2. DNA Extraction

Genomic DNA was successfully extracted from the 282 tumor tissues using the TIAN-amp Genomic DNA kit (Tiangen, Beijing, China).

### 2.3. Selection of CIMP Markers

The “classic panel” of CIMP included* MINT1*,* MINT2*,* MINT31*,* CDKN2A (p16),* and* MLH1*; CIMP was defined as two or more markers methylated [9]. In 2006, a CIMP with new five-gene panel that included* CACNA1G*,* IGF2*,* NEUROG1*,* RUNX3,* and* SOCS1* was defined as three or more markers methylated [36]. At present, there is no gold standard with respect to gene panels and the number of marker thresholds used to define CIMP [37]. Based on the above two CIMP panels and previous studies [38–40], we used seven genes, which mainly used the classic panel as CIMP markers, which included* MINT1*,* MINT31*,* p16*,* MLH1*,* MGMT*,* APC,* and* RUNX3*.

### 2.4. Methylation Sensitive High Resolution Melting (MS-HRM)

Genomic DNA was modified with sodium bisulfite using the EZ Methylation Gold Kit (Zymo Research, Orange, CA). High resolution melting (HRM) was used to assess the methylation status of the seven CIMP-specific promoters (*MLH1*,* MGMT*,* p16*,* APC, MINT1*,* MINT31*, and* RUNX3*). The primers are listed in Table 1. HRM was performed using the following protocol: (1) PCR amplification protocol: denaturation for 10 min at 95°C for 1 cycle, denaturation for 10 s at 95°C, annealing for 30 s at 50°C to 55°C, and extension for 30 s at 72°C for 45 cycles, followed by (2) high resolution melting protocol: 95°C for 1 min, 40°C for 1 min, 74°C for 5 s, and continuous acquisition to 90°C at 25 acquisitions per 1°C (LightCycler 480, Roche, Mannheim, Germany). Each sample was duplicated for two plates. Human methylated and unmethylated DNA sets from Zymo Research were used as 100% methylated and 0% unmethylated controls. The percentages of methylation of 0%, 1%, 5%, 25%, 50%, and 100% were used to draw the standard curve. 5% of methylation was used as cutoff value.

Three levels of CIMP were identified as follows: high-level CIMP (CIMP-H), generally defined as ≥4/7 methylated markers using the seven-marker CIMP panel; low-level CIMP (CIMP-L), generally defined as ≤3/7 methylated markers; CIMP-0, generally defined as 0/7 methylated markers.

### 2.5. Statistical Analysis

The end point was overall survival, calculated from the first diagnosis of colorectal cancer to the death from any cause or until March 2012. The survival curves were estimated using Kaplan-Meier product-limit method. Cumulative survival probability was calculated at the third, the fifth, and the seventh years, respectively. Proportional hazards-regression models were fitted with computing hazard ratios (HR) and the corresponding 95% confidence intervals (95% CI). All statistical tests were 2 sided; *P* values <0.05 were considered statistically significant. All the statistical analysis was performed by SAS 9.1 (SAS Institute, Cary, NC, USA).

## 3. Results

### 3.1. Characteristics of CRC Patients

The study population consisted of 117 females and 165 males, with a mean age of 58.8 ± 11.2 years (range: 25 to 81 years). The median follow-up time was 53 months (range: 1 to 88 months). During the followup, 100 patients died, and 18 patients were lost to followup. The five-year survival of the 282 CRC patients was 58%.

### 3.2. CIMP-Specific Promoter Methylation

CIMP-specific promoter methylation was successfully assessed in all cases. Methylation frequencies in the 282 patients were 25.18% for* MLH1* (71 cases), 34.75% for* MGMT* (98 cases), 19.86% for* P16* (56 cases), 26.60% for* APC* (75 cases), 20.92% for* MINT1* (59 cases), 48.23% for* MINT31* (136 cases), and 19.15% for* RUNX3* (54 cases). 12.77% (36/282) of patients were CIMP-0, 74.1% (209/282) of patients were CIMP-L, and 13.12% (37/282) of patients were CIMP-H.

22.22% (8/36) of CIMP-H tumors were poorly differentiated, while 6.41% (15/234) of CIMP-L/0 tumors were poorly differentiated (*P* = 0.01). In tumor size larger than 5 cm, 60.00% (21/35) were CIMP-H, whereas 36.45% (78/214) were CIMP-L/0 (*P* = 0.01). CIMP-H tumors were more likely to be in TNM III-IV stages than CIMP-L/0 tumors, 63.89% (23/36) versus 43.80% (106/242) (*P* = 0.02). No significant difference was observed in other clinicopathological characteristics (gender, age, tumor location, and histological types) among the CIMP phenotypes. Details are shown in Table 2.

### 3.3. Survival Analysis

#### 3.3.1. Overall Survival Analysis on Clinical and Pathological Status

In multivariate Cox proportional hazards-regression analysis, differentiation and tumor stage were significantly associated with the prognosis of colorectal cancer. Differentiation and TNM stage were adjusted in the following survival analysis.

#### 3.3.2. Gene-Specific Promoter Methylation and Overall Survival

**Figure 1:**
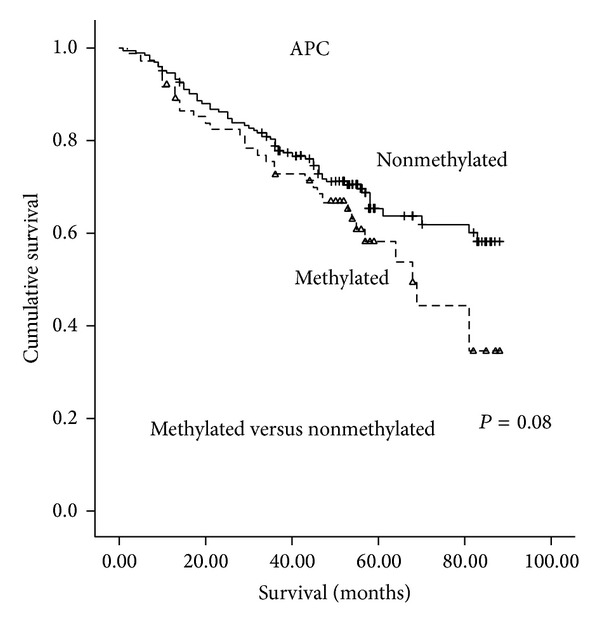
Overall survival according to* APC* methylation levels in colorectal cancer tissue.

When analyzing the associations between gene-specific promoter methylation and overall survival of CRC, there was significant association between* APC *gene promoter methylation and CRC overall survival (HR = 1.61; 95% CI: 1.05–2.46; *P* = 0.03; Figure 1). There was no significant association between other six-gene promoter methylations and CRC prognoses (Table 3).

#### 3.3.3. CIMP Status and Overall Survival

**Figure 2:**
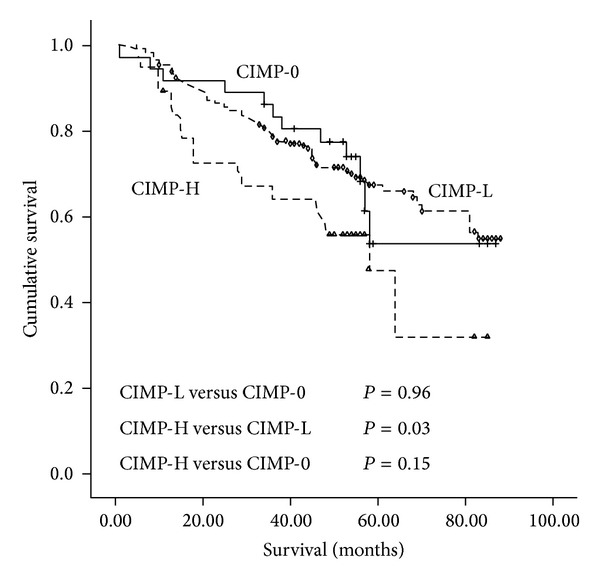
Survival curves in three molecular subgroups according to status of CpG island methylator phenotype (CIMP) in colorectal cancer tissue.

Compared to CIMP-0, CIMP-H was significantly associated with worse prognosis of CRC (HR = 3.06; 95% CI: 1.19–7.89; *P* = 0.02). CIMP-H was also significantly associated with poor prognosis of CRC compared with CIMP-L (HR = 1.97; 95% CI: 1.11–3.48; *P* = 0.02). CIMP-H was also significantly associated with worse prognosis of CRC comparing to the combine of CIMP-L and CIMP-0 (CIMP-L/0) (HR = 2.31; 95% CI: 1.02–5.24; *P* = 0.04) (Table 3, Figure 2).

When analyses stratified according to tumor stage, CIMP-H CRC patients demonstrated a marginally worse prognosis than CIMP-0 CRC patients in stages III to IV group (HR_adj_ = 1.67; 95% CI: 1.00–2.81; *P* = 0.05). However, when comparing to CIMP-L, CIMP-H was not significantly associated with CRC prognosis in stages III-IV group (*P* = 0.20). When comparing to CIMP-L/0, CIMP-H was also not significantly associated with CRC prognosis in stages III-IV group (*P* = 0.07). In stages I-II group, there were no significant differences between the CIMP-H CRC patients and the CIMP-L, CIMP-0, and CIMP-L/0 CRC patients in survival, respectively. Details are shown in Table 4.

A total of 48 (46.2%) of 104 stage III and 38 (21%) of 115 stage II patients received chemotherapy after curative resection of tumor. In CIMP-H patients, 5-FU based adjuvant chemotherapy did not significantly improve overall survival of CRC (HR = 0.71; 95% CI: 0.20–2.54; *P* = 0.60). Patients also did not benefit from 5-FU based adjuvant chemotherapy in CIMP-L/0 group (HR = 1.09; 95% CI: 0.65–1.82; *P* = 0.75).

## 4. Discussion

Epigenetic aberrations are thought to be an important mechanism in human carcinogenesis [41]. One of the epigenetic regulations influencing gene expression is DNA methylation, a postreplicative DNA modification that occurs in genome regions rich in cytosine and guanosine (CG) dinucleotides that are called CpG islands. Modification of bases by addition of a methyl group can physically inhibit binding of transcription factors and also permit recruitment of the methyl-CpG-binding domain proteins to promote regions, which can repress transcription initiation [42]. A subset of colon cancers exhibits widespread promoter methylation, referred to as the CpG island methylator phenotype (CIMP) [43–46]. CRC tumors characterized by CIMP are thought to arise via the serrated neoplasia pathway [47]. An early event in CIMP tumors appears to be a mutation in the* BRAF* protooncogene, which inhibits normal apoptosis of colonic epithelial cells [48]. CRCs with high-level CIMP present distinct clinicopathological and molecular profiles [9, 10]. Clinically, there is evidence to suggest that CIMP-H patients had a shorter cancer-specific survival compared with CIMP-0 patients in CRC [14]. However, another study indicated that CIMP-H appears to be an independent predictor of a low colon cancer-specific mortality [33].

The CpG island methylation phenotype (CIMP), characterized by the extensive hypermethylation of multiple gene CpG islands, was originally described in 1999 by Toyota et al. [49], who defined a subgroup of cancers with a high rate of methylation. Initially, CIMP-positive group was defined as methylation at three or more of seven MINT markers [50]. Subsequently, the so-called “classic panel,” which includes* MINT1*,* MINT2*,* MINT31*,* CDKN2A (p16),* and* MLH1*, was described by Issa in 2004; CIMP-H was defined by high level of methylation at two or more markers [9]. In 2006, Weisenberger et al. [36] introduced a new five-gene panel, which includes the genes* CACNA1G*,* IGF2*,* NEUROG1*,* RUNX3,* and* SOCS1*; CIMP-H was defined by high level of methylation at three or more markers.

At present, many different combinations of gene panel/marker thresholds/laboratory methods were used to define CIMP. Moreover, there is also no standard protocol for choosing primers and/or location of methylation in the markers [37]. The location of core regions and the density of methylation required for gene silencing can vary in every gene; therefore, the classical dogma of promoter CpG island methylation and gene silencing may not be sufficient to interpret data on DNA methylation, gene expression, and clinicopathological associations [51]. Therefore, we used seven genes which mainly used the classic panel as CIMP markers, which included* MINT1*,* MINT31*,* p16*,* MLH1*,* MGMT*,* APC,* and* RUNX3*. A high-throughput platform MS-HRM protocol [52] was used to screen for methylation changes. 0.1% difference can be detected by the high sensitive MS-HRM, and 0.1–10% [53–57] have been used as a cutoff for the scoring criteria of gene methylation. Therefore, 5% of methylation was used as cutoff value. Furthermore, there is debate whether CIMP should be distinguished as two categories (“CIMP-positive” and “CIMP-negative”) [36, 49] or three categories (either “CIMP-H, CIMP-L, or CIMP-0”) [58]. Nosho et al. [59], using a large population-based sample, demonstrated that CIMP-H was independently associated with MSI-high and* BRAF *mutation. Moreover, researchers discovered that when* KRAS* mutation was found in CIMP CRCs, it is associated with CIMP-L. Accumulating evidence suggests that CIMP-L represents a distinct phenotype in CRC [12, 60, 61]. Thus, we used CIMP classification as three categories (either “CIMP-H, CIMP-L, or CIMP-0”).

In our study, we observed significant associations between* APC* promoter methylation and CRC overall survival. CIMP-H was significantly associated with poor CRC prognosis, when comparing to CIMP-L, CIMP-0, and CIMP-L/0, respectively. CIMP-H was a marginally worse prognosis in stages III+IV group, when comparing to CIMP-0 CRC patients.

As to gene-specific promoter methylation,* APC* promoter methylation increased the hazard risk of CRC by 1.61-fold in our study. Hypermethylation of* APC* may provide an important mechanism for impairing* APC* function and could be involved in the progression of human CRC [62, 63]. However, the* APC* promoter methylation demonstrates significantly lower hazards for CRC death (HR = 0.43; 95% CI: 0.19–0.96; *P* = 0.04) in a Taiwanese study [64]. The different methylation detection method may explain the discrepancy. Methylation-specific PCR (MSP) was used in the Taiwanese study, which has a higher false positive rate than MS-HRM [65]. However, there was no significant association between other individual gene methylations and overall survivals of CRC in our study.

Our study investigated that CIMP-H could increase the hazard risk of CRC by 1.97-fold, 3.06-fold, and 2.31-fold, when comparing to CIMP-L, CIMP-0, and CIMP-L/0, respectively. Other eight studies also observed a significant association between CIMP-H and worse CRC prognosis (the HR varied from 1.45 to 5.5) [5, 6, 12–17]. However, one study reported an inverse association between CIMP-H and colon cancer-specific mortality [33]; 15 studies reported a null association between CIMP-H and CRC prognosis [18–32]. However, no clear biological cause for CIMP has been determined, so comparing results across studies is a challenge [37]. The discrepancy might be due to the differences in patient cohorts, as well as the following variation. First, the differences in methylation markers and criteria for CIMP may result in inconsistence. Among the published papers, five papers [12, 18, 20, 28, 66] selected the classical panel of CIMP, where ≥2/5 or 4/5 was defined as CIMP-H; six papers [5, 16, 22, 25, 66, 67] selected the new panel of CIMP, where ≥3/5 was defined as CIMP-H. Two papers [13, 32] selected the classical panel and other genes, where ≥3/*n* was defined as CIMP-H; seven papers [14, 15, 17, 19, 26, 30, 33] selected the new panel and other genes, where ≥5/8 or 6/8 was defined as CIMP-H. Two papers [5, 6] selected methylation markers according to their own criteria, where ≥1/4 or 2/3 was defined as CIMP-H. Meanwhile the variance in the primers and/or location of methylation in the markers may also affect the result. For example, the CIMP marker panel used in Lee et al. study [20] and Barault et al. study [12] was a classic five-marker panel (*MLH1*,* MINT1*,* MINT2*,* MINT31*, and *p16*); different primers of* MLH1*,* MINT1*, and* MINT2* may explain differences of the prognosis of CIMP+ tumors between two studies. In addition, variance in the laboratory method and criteria of methylation could be a reason for the inconsistency of these results of the studies. MSP was used in 12 papers [5, 6, 12, 16, 18, 20, 21, 23, 25, 28, 32, 66]; methylation was assessed by the positive bands in the agarose electrophoresis. MethyLight was used in 10 papers [5, 14, 17, 19, 22, 27, 29, 30, 33, 67]; the percentage of methylated reference (PMR) was used in classification; if the PMR was greater than 4 or 10, it was considered methylated. Bisulfite pyrosequencing was used in 3 papers [13, 24, 68]; each marker was classified as methylated when the mean percentage was higher than 5% or 10%. Moreover, the variable inclusion of other potential confounders in multivariate analysis models may affect the results. Because of the different methylation markers and criteria for CIMP, we could not systematically analyze the association between CIMP status and CRC prognosis upon meta-analysis to obtain a stable result.

When analyses stratified by tumor stage, CIMP-H increased the hazard risk of CRC by 1.67-fold, when comparing to CIMP-0 in stages III-IV group (*P* = 0.05). However, when comparing to CIMP-L, CIMP-0, and CIMP-L/0, respectively, CIMP-H was not associated with worse prognosis of CRC in stages III-IV group. In stages I-II group, there were no significant associations between CIMP and CRC prognosis. One study in Korea yielded a consistent significant association between CIMP-H and worse disease free survival of stage III proximal CRC (*P* = 0.015) [13]. The other study in Germany also demonstrated a significant association between CIMP-H and worse disease free survival of stage II/III rectal cancer (HR = 5.5; 95% CI: 2.1–13.9) [16]. Another study in the USA found that CIMP-H cancers experienced a nonsignificantly low colon cancer-specific mortality in stage III and stage IV colon cancers, respectively (HR = 0.52 (95% CI: 0.17–1.59) for stage III; HR = 0.47 (95% CI: 0.18–1.21) for stage IV) [33]. Stages III and IV are strong determinants for CRC prognosis; CIMP-H was more frequent in stages III and IV CRC than in stages I and II CRC, which may explain the significant association between CIMP and CRC prognosis in stages III and IV CRC. The variant CIMP panels as well as the different cancer sites and different cancer stages may explain the discrepancy.

CIMP-H has been proposed to be having potential application for 5-FU based chemotherapy treatment response prediction [6, 24]. Therefore, we conducted the stratified analyses by 5-FU based chemotherapy. However, we failed to observe any significant association between CIMP-H and the prognosis of stages II+III CRC treated with 5-FU based chemotherapy. A large-scale study also failed to demonstrate a significant association between CIMP-positive and the overall survival of stages II+III CRC treated with adjuvant chemotherapy [32], while CIMP-H was reported to be an independent predictor of survival benefit from 5-FU adjuvant chemotherapy in stage III CRC in another study in Australia [6]. Different CIMP panels and different stages of CRC may explain the discrepancy.

In conclusion, CIMP-H may be a predictor of poor prognosis of CRC, especially for stages III+IV CRC.* APC *gene promoter methylation indicated a poor prognosis of CRC.

## Figures and Tables

**Table 1:** HRM primers and amplicon information.

Gene name	Primer sequences 5′-3′	GenBank	Number of CpG-sites/length
		Accession number	of amplified fragment
*MGMT *	Fw GCGTTTCGGATATGTTGGGATAGT Rv AACGACCCAAACACTCACCAAA	X61657	15/110

*MLH1 *	Fw TTTTTTTAGGAGTGAAGGAGG Rv AACRCCACTACRAAACTAAA	AY217549	13/123

*APC *	Fw AAGTAGTTGTGTAATTCGTTGGAT Rv CACCTCCATTCTATCTCCAATA	NT_034772	11/149

*p16 *	Fw GGAGTTTTCGGTTGATTGGTTGGTT Rv AACAACGCCCGCACCTCCTCTA	AF527803	5/69

*MINT1 *	Fw GGGGTTGAGGTTTTTTGTTAG Rv AATCCCTCTCCCCTCTAAACTT	AF135501	7/137

*MINT31 *	Fw GGGTGATGGTTTTAGTAAAGTGAG Rv AAAAACACTTCCCCAACATCTAC	AF135531	10/164

*RUNX3 *	Fw TTTTTAGAGAATGAGGGATTTTTGT Rv CCCTAATCCCTTAAATCTAATACCC	NC_000001	7/115

**Table 2:** Patient characteristics and phenotypic features of colorectal cancers in tissue by CIMP status.

Variable	Number (*N* = 282)	CIMP-L/0 number (%) (*N* = 245)	CIMP-H number (%) (*N* = 37)	*P*
Age				0.77
<60	146	126 (51.43)	20 (54.05)	
≥60	136	119 (48.57)	17 (45.95)	
Gender				0.10
Female	117	97 (39.59)	20 (54.05)	
Male	165	148 (60.41)	17 (45.95)	
Location				0.22
Proximal	54	42 (17.87)	12 (25.53)	
Distal	228	193 (82.13)	35 (74.47)	
Differentiation				0.01^§^
Poorly	23	15 (6.41)	8 (22.22)	
Moderately or well	247	219 (93.59)	28 (77.78)	
Mutinous				0.91
Yes	59	51 (20.82)	8 (21.62)	
No	223	194 (79.18)	29 (78.38)	
Tumor sizes (cm)				0.01
<5	150	136 (63.55)	14 (40.00)	
≥5	99	78 (36.45)	21 (60.00)	
TNM stage				0.02
I-II	149	136 (56.20)	13 (36.11)	
III-IV	129	106 (43.80)	23 (63.89)	

^§^Fisher exact test.

**Table 3:** Survival analysis on CRC patients according to methylation of individual methylation marker and CIMP status in tumor tissue.

	Number of patients	Number of deaths	Overall survival (%)			Univariate		Multivariate^§^	
			3 y	5 y	7 y	HR and 95% CI	*P*	HR and 95% CI	*P*
*MLH1 *									
Unmethylation	211	76	71.00	56.00	48.00	Ref.		Ref.	
Methylation	71	24	69.00	64.00	64.00	0.94 (0.59–1.48)	0.77	1.07 (0.66–1.74)	0.79
*MGMT *									
Unmethylation	184	65	72.00	59.00	50.00	Ref.		Ref.	
Methylation	98	35	68.00	56.00	56.00	1.02 (0.68–1.54)	0.93	1.05 (0.69–1.61)	0.81
*p16 *									
Unmethylation	226	79	72.00	60.00	53.00	Ref.		Ref.	
Methylation	56	21	67.00	47.00	47.00	1.27 (0.78–2.05)	0.34	1.05 (0.64–1.73)	0.85
*APC *									
Unmethylation	207	67	72.00	63.00	59.00	Ref.		Ref.	
Methylation	75	33	67.00	44.00	34.00	1.46 (0.96–2.21)	0.08	1.61 (1.05–2.46)	0.03
*MINT1 *									
Unmethylation	223	76	73.00	59.00	51.00	Ref.		Ref.	
Methylation	59	24	62.00	53.00	53.00	1.36 (0.86–2.16)	0.19	1.38 (0.85–2.24)	0.20
*MINT31 *									
Unmethylation	146	52	72.00	56.00	50.00	Ref.		Ref.	
Methylation	136	48	69.00	59.00	54.00	1.04 (0.70–1.54)	0.84	1.01 (0.67–1.53)	0.96
*RUNX3 *									
Unmethylation	228	83	70.00	58.00	51.00	Ref.		Ref.	
Methylation	54	17	73.00	55.00	55.00	0.90 (0.53–1.52)	0.69	1.14 (0.67–1.95)	0.63
CIMP status									
CIMP-0	36	12	77.00	59.00	59.00	Ref.		Ref.	
CIMP-L	209	70	72.00	61.00	54.00	0.99 (0.53–1.82)	0.96	0.95 (0.60–1.52)	0.84
CIMP-H	37	18	58.00	33.00	33.00	1.71 (0.82–3.55)	0.15	3.06 (1.19–7.89)	0.02
CIMP-H versus CIMP-L						1.78 (1.07–3.03)	0.03	1.97 (1.11–3.48)	0.02
CIMP-H versus CIMP-L and CIMP-0						1.80 (1.08–3.00)	0.03	2.31 (1.02–5.24)	0.04

^§^Multivariate analysis, adjusted for age at diagnosis, tumor stage, tumor differentiation, and tumor location.

**Table 4:** Survival analysis on CRC patients according to CIMP status in tumor tissue in different tumor stages.

CIMP status	Stages I-II (*n* = 149)				Stages III-IV (*n* = 129)			
	Univariate		Multivariate^§^		Univariate		Multivariate^§^	
	HR and 95% CI	*P*	HR and 95% CI	*P*	HR and 95% CI	*P*	HR and 95% CI	*P*
CIMP-H versus CIMP-L/0	0.69 (0.17–2.91)	0.62	0.52 (0.12–2.22)	0.38	1.81 (1.02–3.24)	0.04	1.75 (0.95–3.23)	0.07
CIMP-H versus CIMP-L	0.74 (0.17–3.14)	0.68	0.57 (0.13–2.48)	0.46	1.73 (0.96–3.11)	0.07	1.50 (0.80–2.81)	0.20
CIMP-L versus CIMP-0	0.66 (0.27–1.60)	0.35	0.57 (0.23–1.41)	0.22	1.42 (0.60–3.34)	0.43	2.06 (0.77–5.53)	0.15
CIMP-H versus CIMP-0	0.71 (0.32–1.58)	0.40	0.56 (0.21–1.49)	0.24	1.55 (0.96–2.49)	0.07	1.67 (1.00–2.81)	0.05

^§^Multivariate analysis, adjusted for age at diagnosis, tumor stage, tumor differentiation, and tumor location.
